# Can orthopedic trials change practice? A survey of 796 orthopedic surgeons on the possible findings of a hip fracture trial

**DOI:** 10.3109/17453671003587093

**Published:** 2010-03-31

**Authors:** Bernadette G Dijkman, Bauke W Kooistra, Julia Pemberton, Sheila Sprague, Beate P Hanson, Mohit Bhandari

**Affiliations:** ^1^Division of Orthopedic Surgery, Departments of Surgery and Clinical Epidemiology and Biostatistics. McMaster University, Hamilton, OntarioCanada; ^2^AO Clinical Investigation, ZurichSwitzerland

## Abstract

**Background and purpose:**

The impact of large, randomized trials in orthopedic surgery on surgeons' preferences for a particular surgical approach remains unclear. We surveyed surgeons to assess whether they would change practice based upon results of a large, multicenter randomized controlled hip fracture trial.

**Methods:**

We conducted a cross-sectional survey among International Hip Fracture Research Collaborative (IHFRC) surgeons and surgeons who were members of Arbeitsgemeinschaft fuer Osteosynthesefragen - Association for the Study of Internal Fixation (AO/ASIF) to determine the likelihood that they would change practice based on findings of a proposed large, multicenter randomized controlled trial (the Hip Fracture Evaluation with Alternatives of Total Hip Arthroplasty versus Hemi-Arthroplasty (HEALTH) study). We asked surgeons their current preferences for the management of displaced femoral neck fractures and whether a trial that definitively revealed a substantial improvement in function and quality of life with no difference in risk of revision surgery was important and would cause them to change practice.

**Results:**

Of 883 surgeons surveyed, 210 responded from IHFRC and 586 from AO/ASIF (a response rate of 90%). Most surgeons (61%) preferred hemiarthroplasty (HA) for treating displaced femoral neck fractures. 72% of responding surgeons believed that a substantial improvement in patient function with total hip arthroplasty (THA) and no adverse effects on revision surgery would be an important finding. Moreover, of 483 surgeons who preferred hemiarthroplasty, 62% would change their practice based upon the findings of the trial.

**Interpretation:**

Large clinical trials in orthopedics are worthwhile endeavors, as they have the potential to change practice among surgeons. Surgeons seem willing to adopt alternative surgical approaches if the evidence is compelling and sound.

## Introduction

Evidence-based medicine (EBM) is “the process of systematically finding, appraising, and using contemporaneous research findings as the basis for clinical decisions” (Rosenberg and Donald 1995). According to this principle, clinical decisions should mainly be guided by the results of the best available evidence. At the top of the hierarchy of evidence is the randomized controlled trial (Brighton et al. 2003).

Despite the increased enthusiasm for EBM, it is questionable whether surgeons' preferences for a particular surgical approach can be influenced by high-quality evidence. A French survey showed that although 75% of surgeons said they used results from randomized controlled trials, only 25% could provide the results of a randomized controlled trial that had actually changed their practice (Millat et al. 1999). In addition, for 58% of English hospital physicians, evidence from EBM sources contributed to changes in their clinical practice (Coleman and Nicholl 2001).

The extent to which a surgeon puts EBM into practice could be lower for surgeons who are not very research-conscious or interested in research (Kizer et al. 1999), or for surgeons who do not have enough knowledge about or experience of EBM (McAlister et al. 1999). Secondly, the perceived importance of outcomes might influence the implementation of the results. Given that conduction of large, randomized trials in orthopedic surgery requires a large investment of funding, one could argue that only studies that have the potential to change surgical practice should receive funding priority.

Conflicting evidence (Blomfeldt et al. 2007, Goh et al. 2008, Macaulay et al. 2008) and expert opinion (Bhandari et al. 2005) about the optimal treatment of displaced (Garden III–IV) (Garden 1961) femoral neck fractures have led to conduction of a pilot multinational randomized trial entitled “Hip Fracture Evaluation with Alternatives of Total Hip Arthroplasty versus Hemi-Arthroplasty” (HEALTH). This trial is evaluating the feasibility of a large, multicenter randomized controlled trial comparing THA and HA regarding revision surgery, function, and quality of life in 2,500 otherwise healthy elderly patients with displaced femoral neck fractures.

The purpose of the present survey was to assess whether orthopedic surgeons would change practice based on a large, multicenter randomized controlled trial if it revealed a significant improvement in patient-important outcomes. We also tried to explore the difference between surgeons with an interest in research and those without any research interest.

## Methods

This survey of surgeons was based on an ongoing randomized trial evaluating alternative arthroplasty approaches in patients with displaced femoral neck fractures.

### The HEALTH randomized trial

The HEALTH randomized controlled trial asks the primary question: In patients over 50 years who have sustained a displaced femoral neck fracture, what is the rate of revision surgery at 2 years in those treated with THA as opposed to HA? Study personnel monitor critical aspects of perioperative care and rehabilitation for protocol deviations. In the pilot study, the investigators are evaluating a novel expertise-based design, screening procedures, consent rates, adherence to study protocols, and follow-up rates with a view to streamlining these procedures. The primary outcome is revision surgery within 2 years of surgery. The secondary outcomes include patient function (Western Ontario McMaster osteoarthritis index (WOMAC), timed up-and-go test (TUG)), quality of life (SF-12 version 2), and health utility (EuroQol-5D (EQ-5D)). Investigators will independently adjudicate revision surgery rates at regular intervals up to 2 years. Funding has been received from the Canadian Institutes of Health Research (CIHR) and the National Institute of Arthritis, Musculoskeletal, and Skin Diseases (NIAMS) within the National Institutes of Health (NIH) to begin a pilot study at 17 centers in the United States, Canada, Europe, and Australia. The HEALTH pilot study will include 156 pilot patients to provide information on the feasibility of a larger, definitive trial involving 2,500 patients. In addition, funding has been received from the ZonMw, the Netherlands Organization for Health Research and Development, for 7 centers and 150 patients in the Netherlands. HEALTH began recruitment in December 2008 and as of early September 2009, 40 patients have been enrolled across 13 centers screening for patients. The hypothesis is that total hip arthroplasty will have similar or lower rates of revision surgery (i.e. within 5% of each other) and higher functional outcome scores (i.e. 6 point difference in SF-12 version 2) at 2 years compared to hemiarthroplasty.

### Survey design

We conducted a cross-sectional survey among International Hip Fracture Research Collaborative (IHFRC) surgeons (www.ifrca.ca) and AO/ASIF membership surgeons to determine the likelihood that they would change practice based on the findings of our proposed large, multicenter randomized controlled trial (the HEALTH study). The IHFRC is a collaboration of surgeons who are interested in participating in large trials on the optimal treatment of hip fracture.

The survey involved 3 key areas. First, the participants were asked about their current preferences for the management of displaced femoral neck fractures in otherwise healthy elderly patients (THA, HA, or internal fixation). Next, the surgeons were asked whether they (1) would find it important and (2) would change their surgical practice if the HEALTH trial revealed that THA was superior regarding pain, function, and quality of life (QoL)), with no difference in risk of revision surgery. The response options for the latter 2 questions were presented as Likert scales. Superiority was defined as significantly reduced pain score with total hip arthroplasty (p < 0.05) and significantly improved functional score (SF-12 version 2, 6 points). We defined lack of inferiority of total hip replacement as being within 5% of the revision surgery rate of hemiarthroplasty at 2 years.

### Survey administration

We identified all active orthopedic surgeons who were members of the IHFRC and all surgeons with AO/ASIF membership. The survey was sent to each participant by both e-mail and fax, and was accompanied by a personalized cover letter explaining the purpose of the survey. At 2, 4, 8, and 12 weeks after the initial mailing, we re-sent the survey to all non-responders. Individual responses remained confidential, and completion of the questionnaire was voluntary.

### Data analysis

We summarized frequencies of the responses as percentages. All answers were included in the analyses, irrespective of whether the entire questionnaire was completed or not. For comparative analysis between AO/ASIF and IHFRC members, the chi-square test was used. Results with a p-value of < 0.05 were considered significant.

## Results

Of the 883 surgeons surveyed, 210 from IHFRC responded and 586 from AO/ASIF responded (giving a response rate of 90%) (Table 1). Most surgeons (n = 483, 61%) preferred hemiarthroplasty for treating displaced femoral neck fractures (Table 2). Notably, more IHFRC members preferred HA than AO/ASIF members (74% vs. 56%, p < 0.001).

**Table 1.** Geographical location of the respondents (n = 796)

Continent of practice	Number (%) of respondents
Europe	361 (45)
North America	161 (20)
Asia	132 (17)
South America	41 (5.2)
Africa	11 (1.4)
Not answered	90 (11)

**Table 2.** Management preferences of the respondents (n = 796)

	Number (%) of respondents		
Intervention	IHFRC (n = 210)	AO/ASIF (n = 586)	Total (n = 796)
Hemiarthroplasty	155 (74)	328 (56)	483 (60)
Total hip arthroplasty	20 (9.5)	96 (16)	116 (15)
Internal fixation	10 (4.8)	129 (22)	139 (18)
No single preference	19 (9.1)	0 (0.0)	19 (2.3)
Not answered	6 (2.9)	33 (5.6) **^a^**	39 (4.9)
**^a^** p < 0.001 for differences between proportions across IHFRC and AO/ASIF members.			

72% of the surgeons who responded believed that a substantial improvement in patient function and less pain with THA, with similar revision surgery rates to HA, would be an important finding (definitely important: 32%; possibly important: 40%) (Table 3). A higher proportion of IHFRC members considered such findings to be definitely important compared to AO/ASIF members (67% vs. 20%, p < 0.001).

**Table 3.** Perceived importance to orthopedic practice of patient-important findings ^a^ that would support total hip arthroplasty

	Number (%) of respondents		
Perceived importance	IHFRC (n = 210)	AO/ASIF (n = 586)	Total (n = 796)
Definitely important	141 (67)	116 (20)	257 (32)
Possibly important	55 (26)	261 (45)	316 (40)
Total (important)	196 (93)	377 (64)	573 (72)
Possibly unimportant	4 (1.9)	43 (7.3)	47 (5.9)
Definitely unimportant	2 (1.0)	19 (3.2)	21 (2.6)
Total (unimportant)	6 (2.9)	62 (11)	68 (8.5)
Unsure	3 (1.4)	89 (15)	92 (12)
Not applicable	0 (0.0)	2 (0.3)	2 (0.3)
Not answered	5 (2.4)	56 (9.6) **^b^**	61 (7.7)
**^a^** In terms of reduced pain and improved patient function and quality of life.			
**^b^** p < 0.001 for differences between proportions across IHFRC and AO/ASIF members.			

Of 483 surgeons who preferred HA for the management of displaced femoral neck fractures in the elderly, 62% responded that they would change their practice based upon the findings of a trial reporting superiority of THA (Table 4). Of those, 13% would definitely, 26% would very likely, and 24% would be somewhat likely to change their practice from HA to THA (Table 4). Surgeons who were members of AO/ASIF and preferred HA were less likely to change their practice than surgeons who were members of the IHFRC (48% vs. 92%, p < 0.001).

**Table 4.** Likelihood that surgeons using HA would change their practice on the basis of patient-important findings ^a^ that would support total hip arthroplasty

	Number (%) of respondents		
Likelihood of changing	IHFRC (n = 155)	AO international (n = 328)	Total (n = 483)
Definitely	49 (32)	13 (4.0)	62 (13)
Very likely	65 (42)	59 (18)	124 (26)
Somewhat likely	28 (18)	86 (26)	114 (24)
Total (change)	142 (92)	158 (48)	300 (62)
Not likely	7 (4.5)	87 (27)	94 (20)
Never	0 (0.0)	5 (1.5)	5 (1.0)
Total (no change)	7 (4.5)	92 (28)	99 (21)
Uncertain	5 (3.2)	56 (17)	61 (13)
Not applicable	0 (0.0)	1 (0.3)	1 (0.2)
Not answered	1 (0.6)	21 (6.4) **^b^**	22 (4.6)
**^a^** In terms of reduced pain and improved patient function and quality of life.			
**^b^** p < 0.001 for differences between proportions across IHFRC and AO/ASIF members.			

## Discussion

Our findings support the notion that surgeons will change practice if large, high-quality trials suggest that there are important benefits with alternative therapies. Most surgeons in our survey preferred HA for the management of displaced femoral neck fractures. If our findings are correct (i.e. that 62% would switch to THA if a trial confirmed its superiority), the proportion of surgeons using THA as the preferred treatment would increase from 15% (116) to 52% (416). The most conservative estimate of impact of the HEALTH trial, if positive, would increase the proportion of surgeons using THA from 15% (116) to 38% (302).

Whether or not funding of major research by the public or private sector would require assurance of such willingness to change practice remains unknown. Our findings certainly suggest that the results of large trials can have an impact on clinical practice. This emphasizes the widespread need for orthopedic surgeons to support and participate in such trials.

This survey had several limitations. First, only the scenario of superiority of THA over HA with respect to patient function was presented to participants of the survey. Our findings are not generalizable to any other possible study findings. Secondly, it may not be possible to generalize the results of this survey to more complicated cases, as the scenario in the survey assumed that patients were otherwise healthy. In addition, the participants of this survey might not represent orthopedic surgeons in general, since all participating surgeons were members of an organization of orthopedic surgery and one could question whether non-members might respond as often to surveys as members. Furthermore, hip fractures in the elderly are associated with a relatively high degree of reduction of function compared to other fractures, which makes it less useful to apply these results to trials comparing treatments of other fractures. However, patient-important outcomes are likely to influence all clinical decisions of surgeons and physicians, independently of the treatments compared.

Despite these limitations, our study was strengthened by the high response rate of 90%, which reduces potential non-responder bias. Also, since a sample of average members (AO/ASIF) and a sample of members who are highly research-orientated (IHFRC) were included, the participants of this survey probably represent a cross-section of the overall orthopedic surgery community.

The concept of EBM was first described in the literature in 1991 (Guyatt 1991). Using best evidence, one could expect that surgical decisions would be based on results from large, multicenter randomized controlled trials. Unfortunately, results from randomized controlled trials do not contribute very often to the clinical decision making of both general surgeons and hospital physicians (Millat et al. 1999, Coleman and Nicholl 2001, Hadley et al 2007). Our findings do, however, suggest that orthopedic surgeons may in fact be influenced by high-quality data.

Cardiologists have been shown to be more likely to increase their prescription of drugs that have been demonstrated to be effective in recent randomized controlled trials when they have participated in trials themselves (Kizer et al. 1999). As the IHFRC members are specifically interested in trials on the optimal treatment of hip fractures, they are more likely than AO/ASIF members to indicate that they would change their practice based on the HEALTH trial. Also, one could reason that IHFRC members would consider superiority of THA over HA to be more important than AO/ASIF members, since HA is more frequently used by IHFRC members (74%) than by AO/ASIF members (56%). Our results show that a substantially higher proportion of IHFRC members would change their clinical practice. Furthermore, a higher proportion of IHFRC members would consider the findings to be definitely important as compared to AO/ASIF members. Thus, our results support the hypothesis that research-orientated surgeons would be more likely to change their practice based upon the findings of a large, randomized controlled trial.
